# Population Pharmacokinetics of Enrofloxacin in *Micropterus salmoides* Based on a Nonlinear Mixed Effect Model After Intravenous and Oral Administration

**DOI:** 10.3390/ani15101362

**Published:** 2025-05-08

**Authors:** Ning Xu, Shun Zhou, Jing Dong, Jiangtao Li, Yongzhen Ding, Xiaohui Ai

**Affiliations:** 1 Yangtze River Fisheries Research Institute, Chinese Academy of Fishery Sciences, Wuhan 430223, China; xuning@yfi.ac.cn (N.X.); zhoushun@yfi.ac.cn (S.Z.); dongjing@yfi.ac.cn (J.D.); lijiangtao@yfi.ac.cn (J.L.); 2 Freshwater Fisheries Research Center, Chinese Academy of Fishery Sciences, Wuxi 214081, China; 3 Agro-Environmental Protection Institute, Ministry of Agriculture and Rural Affairs, Tianjin 300191, China

**Keywords:** enrofloxacin, population pharmacokinetics, statistical approaches, largemouth bass, body weight

## Abstract

This study aimed to investigate the PPK of EF in largemouth bass after oral and intravenous administration based on a nonlinear mixed effect model. Samples were collected using the sparse sampling method at pre-designed time points determined by high-performance liquid chromatography with a fluorescent detector. The initial PK parameters were estimated by reference search and the calculation of a naïve pooled approach. The covariate model included a variation in body weight. The oral dose data were best fitted by a one-compartment model. The injection dose data were best fitted by a two-compartment model. The results demonstrated that body weight had no marked effect on the parameters of PPK. Finally, the bioavailability was calculated to be 12.24%. The area under the concentration–time curve/minimum inhibitory concentration was estimated to be ≥408.16, indicating that EF at 20 mg/kg has high effectiveness for aquatic pathogens.

## Simple Summary

Population pharmacokinetics (PPK) is an interdiscipline of pharmacokinetics (PK) and statistics. It reflects the influence of fixed effects and stochastic effects on PK and involves the collection of blood samples using the sparse time points method that solves the problem of insufficient blood volume in fish. Therefore, we tried to simulate the PK parameters of enrofloxacin (EF) in largemouth bass (*Micropterus salmoides*) using the PPK model. This exploration can provide a concise strategy to promote the development of PK methodology for aquatic animals.

## 1. Introduction

Pharmacokinetics (PK) is an interdisciplinary science involving pharmacy and mathematics that can quantitatively characterize the absorption, distribution, metabolism, and excretion of medicine in animals or humans. Since about the 1970s, PK has been used in aquatic animals for describing drug disposition and determining proper dosage regimens [1,2]. Compared to mammals and birds, the limited blood volume of fish means that insufficient blood samples can be drawn from an individual organism. Hence, a discontinuous collection method, drawing samples from different fish at a time point (4–6 animals per point), was developed to conduct these studies [3,4,5,6]. This method overcame the disadvantage of the limited blood volume of fish and enabled enough blood samples to be collected at different time points. Nevertheless, the obtained data included differences derived from fish physiology and biochemistry that caused large variations [7,8]. Abnormal concentration peaks and fluctuations were easily brought into the PK profiles, which led to incorrectly reflecting the properties of drug disposition and even the inability to iterate PK parameters using compartmental models.

Population pharmacokinetics (PPK) is a new discipline combining classic PK and statistics that can investigate the influence of fixed effects and stochastic effects on blood concentration. It has many advantages including the following: (1) it can use sparse samples to simulate the model to estimate PK parameters, thereby reducing the sampling time points and the number of experimental animals; (2) fixed effects (body weight, gender, age, body height, etc.), variation between individuals, and variation within an individual can be used to help fit PK parameters, which facilitates formulating dosage regimens and optimizing routes of medicine administration for special patients [9,10]. These PPK characteristics just solve the problem of not being able to continuously sample in fish groups, and the introduction of fixed effects and stochastic effects makes values of PK parameters more precise for individual medicine administration.

In the present study, we will try to conduct a PPK investigation into enrofloxacin (EF) in aquatic animals using a sparse sampling method. EF is an important medicine in global aquaculture against various pathogen bacteria including *Aeromonas salmonicida*, *Aeromonas hydrophila*, *Aeromonas viridis*, *Edwardsiella ictalurid*, *Yersinia ruckeri*, *Flavobacterium psychrophilum*, etc. [11,12,13,14]. Traditional PK using a continuous collection method has been carried out in Nile tilapia (*Oreochromis niloticus*) [15], brown trout (*Salmo trutta*) [16], largemouth bass (*Micropterus salmoides*) [17], northern snakehead (*Channa argus*) [18], and yellow river carp (*Cyprinus carpio haematoperus*) [19]. Limited information on PPK is available in aquatic animals. We will select a commercially important fish species of largemouth bass (*Micropterus salmoides*), its production reaching 0.82 million tons in 2020 in China [20], to perform these studies after intravenous and oral administrations. This exploration can promote the development of PK methodology in aquaculture and provide a concise strategy for optimizing dosage regimens and modes of administration in aquatic animals.

## 2. Materials and Methods

### 2.1. Chemicals and Reagents

Analytical standard of enrofloxacin (EF, CAS number: 93106-60-6) with a purity of ˃99.2% was bought from Yuanye Biotechnology Co., Ltd. (Shanghai, China). Its molecular formula and molecular weight are C_19_H_22_FN_3_O_3_ and 359.39 Da. Huisheng Biotechnology Co., Ltd. (Wuhan, China) provided the power of EF with a purity of ≥30.0% used for intravenous injection and oral gavage. The reagents were all high-performance liquid chromatography (HPLC) grade including acetonitrile, hydrochloric acid, methanol, water, and formic acid purchased from Maclin Biochemical Technology Co., Ltd. (Shanghai, China). Anhydrous magnesium sulfate was bought from Yuanye Biotechnology Co., Ltd. (Shanghai, China).

### 2.2. Fish Management

Sixty largemouth bass (243.11 ± 54.86 g) were purchased from the culturing facility of the Huazhong Agricultural University and used for intravenous administration. All fish were kept in tanks with a volume of 480 L per tank receiving water (26 L/min). They acclimatized to a new water environment for 14 days and were fed antibiotic-free feed provided by Huihai Formulation Feed Co. Ltd. (Jingzhou, China) [21]. Experimenters determined parameters of water quality every day to ensure that dissolved oxygen levels, total ammonia nitrogen levels, nitrite nitrogen levels, and pH values were in the appropriate ranges [22]. The water temperature was maintained at 25.0 ± 0.5 °C by an air conditioner. The blank plasma was sampled from fish without administering antibiotics and stored at −20 °C, which was utilized to develop an HPLC method for EF. All experimental procedures and protocols were approved by the Fish Ethics Committee of Yangtze River Fisheries Research Institute, Chinese Academy of Fishery Sciences, Wuhan, China (YFI2024xuning01).

### 2.3. EF Exposure and Sampling

#### 2.3.1. Oral Administration

The EF powder was dissolved in pure water to obtain a final concentration of 20 mg/mL. Twenty-four largemouth bass were randomly divided into four groups. Each fish was administered a proper volume of EF solution at a dose of 20 mg/kg in line with body weight by a plastic tube linked to a 2.5 mL microinjector in the stomach. After oral gavage, the fish was put in a separate tank to observe whether the fish regurgitated EF solution. If the EF solution was regurgitated, the fish was removed from the study and replaced.

Each fish was subjected to four sampling time points. In the first group, blood was continuously collected blood from the caudal vessels from each fish at time intervals of 5 min and 1, 8, and 48 h after oral gavage. In the second group, blood was collected from fish after 10 min amd 2, 12, and 72 h. In the third group, blood was drawn from fish at 15 min and 4, 16, and 96 h. In the fourth group, blood was collected from fish at 0.5, 6, 24, and 120 h. Blood samples were immediately centrifugated at 1500× *g* for 5 min to obtain plasma. All samples were preserved at −20 °C until analysis [23,24].

#### 2.3.2. Intravenous Administration

The EF solution with a concentration of 20 mg/mL was prepared using the above-mentioned method. Twenty-four largemouth bass were randomly divided into four groups. Each fish was weighed and given a sufficient volume of EF solution at a dose of 10 mg/kg using a 1 mL microinjector into the caudal vein. If the fish was heavily bleeding after withdrawing the needle or the needle had translocated during injection, the fish was excluded from the study and replaced.

The planning of sampling time points was consistent with oral administration. Blood samples were immediately centrifugated at 1500× *g* for 5 min to obtain plasma. All samples were preserved at −20 °C until analysis.

### 2.4. Sample Preparation

The method of sample preparation was taken from our published paper [25]. Plasma samples were thawed at ambient temperature, and then about 400 µL of plasma was transferred into a 10 mL centrifugation tube. To each tube was added 4 mL of acidified acetonitrile (acetonitrile–hydrochloric acid–water = 250:1:1, *v*/*v*/*v*) and shaken for 30 s. Subsequently, to the tube was added 0.5 g of anhydrous magnesium sulfate with shaking for 30 s to remove moisture in plasma and tissues. After centrifugation for 5 min at 4000× *g*, the resulting supernatant was transferred into a new tube. According to the same procedures, samples were extracted once again, and the supernatants were merged. Afterward, the extracted liquid was evaporated by a slight nitrogen stream at 50 °C until complete dryness. A volume of 1 mL of acetonitrile and 0.2% formic acid water (18:82) was added to reconstruct the residue, and 1 mL n-hexane was added into the mixture to remove fat after being shaken for 30 s. The mixture was further centrifugated for 5 min at 7000× *g*. The lower liquid was filtrated by a 0.22 µm nylon filter for HPLC analysis.

### 2.5. HPLC Analysis and Method Validation

Prepared samples were detected by an Agilent 1260 Infinity II HPLC (Santa Clara, CA, USA) coupled with a fluorescent detector, a quaternary solvent manager with a quaternary solvent pump, and a sampler manager with an autosampler. The EF was separated by a Poroshell 120 EC-C18 column (2.7 μm, 4.6 × 100 mm) with a set temperature of 35 °C. The liquid phase included water containing 0.2% formic acid and pure acetonitrile. An isocratic elution with a proportion of 82:18 was used to run samples. The flow rate was 0.8 mL/min. The excitation and emission wavelengths were set to 280 and 450 nm.

The method validation was performed according to the guidelines of the EU Commission Decision 2021/808/EC [26]. The procedures were described in detail in our previously published papers [25,27].

### 2.6. PPK Parameterization

The initial values of parameters were computed with a classic one-compartment model based on a naïve averaged pooled sample method for concentration profile of oral administration. The PPK analysis was conducted on the Phoenix NLME (Phoenix 8.0) software. We built the basic PK model by selecting a one-compartment model with extravascular administration for oral gavage. The values of parameters and corresponding variation coefficients were calculated using the method of the first-order conditional estimation extended lead squares. An exponential model was used to estimate the inter-individual variability and inter-occasional variability of PK parameters, which was parameterized as below:

Pi = tvP × exp (ηPi)

where Pi is a PK parameter for the ith individual, tvP is the value of the parameter within the population, and ηPi is the random effect. The random effects were assumed to be independent and have a normal distribution with a mean of 0 and a variance of ω^2^. The variability was described as coefficients of variation calculated by the mean of the equation, as follows:

$$\mathrm{CV}\;(\%)=100\times \sqrt{\exp\left(\omega^{2}\right)-1}$$

The multiplicative model was employed to calculate the residual unexplained variability. The related equation is shown below.

OBSV = IPCN × (1 + ε)

where OBSV is the observed value, IPCN is the individual predicted concentration, and ε is normal distribution, which is zero-mean chance variables with a variance.

The goodness of fit of the PPK model was estimated by the diagnostic logarithmic plots of the dependent variable (DV) versus (vs.) individual predicted concentrations (IPRED) and the plot of conditional weighted residuals (CWRES) vs. time after dose (TAD). When the basic model was established, the covariates were cited in the PPK model to evaluate the variation between individuals. The relevant covariates consisted of body weight, gender, blood indicators, urine indicators, etc. For fish species, the body weight was easily obtained. So, the covariate of body weight was chosen to estimate the influence on PPK parameters. Through running different covariate combinations, proper PK parameters adding the influence of body weight were selected by comparing the likelihood ratio test (LRT), the values of −2 × log maximum likelihood (−2LL), the Akaike’s inclusion criteria (AIC), and Bayesian information criterion (BIC).

After oral administration, the initially calculated parameters were the apparent distribution volume (V), absorption rate constant (K_a_), and systemic clearance (CL). Secondary parameters of the elimination rate constant (K_e_), elimination half-life (T_1/2Ke_), and the area under the concentration–time curve (AUC_0–∞_), were evaluated by PK equations, as below.

K_e_ = tvCL/tvV

T_1/2Ke_ = ln2/K_e_

AUC_0–∞_ = Dose/tvCL

The initially evaluated parameters after intravenous administration of CL, the clearance from the central compartment to the peripheral compartment (CL_2_), apparent distribution volume of the central compartment (V_1_), and apparent distribution volume of the peripheral compartment (V_2_) were calculated using a two-compartmental model by a naïve averaged pooled sample method. A two-compartment model with intravenous administration was selected to be the basic structural PK model. Other parameterizations were consistent with the above description.

Secondary parameters included the elimination rate constant (K_β_), elimination half-life (T_1/2β_), the area under the concentration–time curve (AUC_0–∞_), first-order transport rate constant from central compartment to peripheral compartment (K_12_), and first-order transport rate constant from peripheral compartment to central compartment (K_21_), calculated using the PK equations shown below.

K_β_ = tvCL/(tvV1 + tvV2)

K_12_ = tvCL_2_/V_1_

K_21_ = tvCL_2_/V_2_

T_1/2β_ = ln2/K_β_

AUC_0–∞_ = Dose/tvCL

### 2.7. Model Validation

The PK parameters of the final model were validated by bootstrap. Visual inspection of routine diagnostic plots, histograms and QQ figures, and individual plots were used for evaluating the final model.

## 3. Results

### 3.1. HPLC Analysis

The results showed that the values of the limit of detection and limit of quantification were 0.003 µg/mL and 0.01 µg/mL in plasma. The calibration curve equation is y = 0.9408x + 10.871 (R = 0.9999). Table 1 shows the recovery rates, standard deviations of inter-day, and standard deviations of intra-day. The mean recovery rates of EF ranged from 83.29% to 103.12% in plasma. Their percentage relative inter-day standard deviations ranged from 2.11% to 3.21%, and intra-day precision values ranged from 3.01% to 6.72%. In the analysis procedure, if the concentrations of EF were more than the upper limit of quantification in some samples, the remaining samples were diluted with corresponding blank plasma to repeat the measurement.

### 3.2. Initial Estimation

The naïve averaged pooled sample method was used to calculate the initial PK parameters of EF. The concentration data after oral gavage were iterated by the one-compartment model, and the concentration data after intravenous injection were iterated by the two-compartment model. The related values of the parameters are listed in Table 2.

### 3.3. PPK Parameters

**Figure 1:**
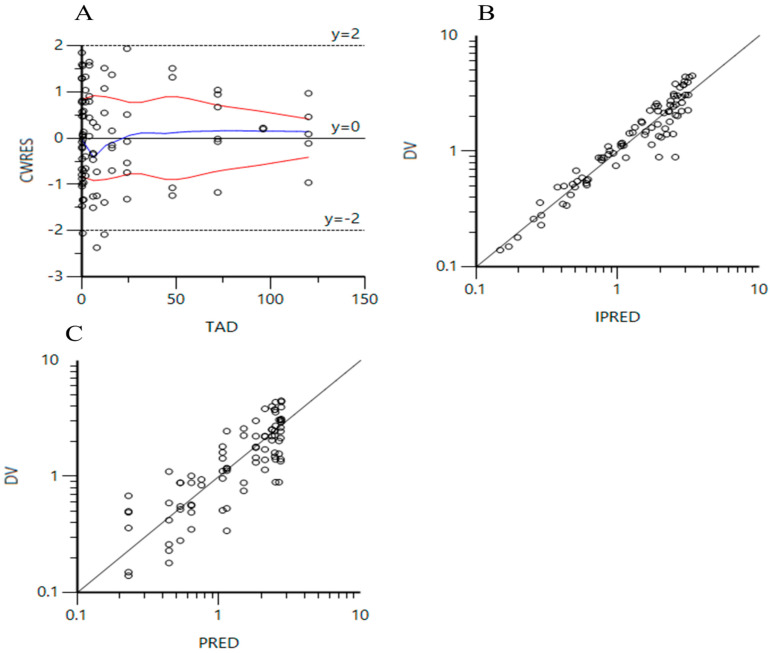
(**A**): Plot of conditional weighted residual values (CWRES) versus time after oral dose (TAD); (**B**): logarithmic plot of the dependent variable (DV) versus individual predicted plasma concentrations (IPRED) of enrofloxacin in all experimental animals; (**C**): logarithmic plot of the dependent variable (DV) versus predicted plasma concentrations (IPRED) of enrofloxacin in all experimental animals.

After building the basic model, initial parameters after oral administration were imported and objective function values (OFVs) under different scenarios were calculated. We found that the body weight has a weak influence on the OFVs. Ultimately, the lowest OFV of 123.11 with the least number of parameters associated with V-wt was selected to iterate the model. The results are listed in Table 3. Compared to the model without the covariate of body weight, the standard error and coefficient of variation (CV) showed minor changes (Table 4). Therefore, the parameter of body weight was not introduced in the final model. The plot of CWRES vs. TAD and logarithmic plots of DV vs. PRED or IPRED showed that the model can be appropriately fitted to the concentration changes in EF in most animals after oral administration (Figure 1). Finally, the values of K_a_, V, and CL were evaluated to be 0.98/h, 6.82 L/kg, and 0.098 L/h/kg, respectively. The secondary parameter values of K_e_, T_1/2Ke_, and AUC_0–∞_ were computed to be 0.014/h, 49.50 h, and 204.08 h.mg/L, respectively.

**Figure 2:**
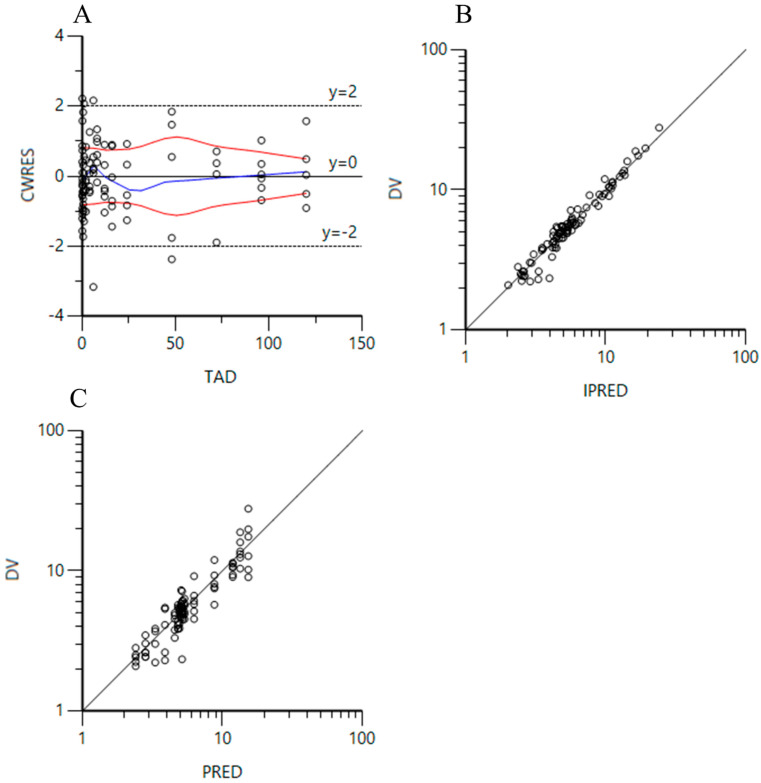
(**A**): Plot of conditional weighted residual values (CWRES) versus time after intravenous dose (TAD); (**B**): logarithmic plot of the dependent variable (DV) versus individual predicted plasma concentrations (IPRED) of enrofloxacin in all experimental animals; (**C**): logarithmic plot of the dependent variable (DV) versus predicted plasma concentrations (IPRED) of enrofloxacin in all experimental animals.

After intravenous injection, Table 5 shows the OFVs in different scenarios. Like the data of oral administration, the covariate of body weight was imported into the model, which did not cause large changes in the parameter. The lowest OFV with the lowest number of parameters associated with V_2_-wt was 270.34, and was selected to fit the PPK model (Table 5). The results showed that the standard error and coefficient of variation of the simulated PK parameters displayed minor changes, and the dV_2_dwt (body weight effect on V_2_) was estimated to be a minus value (Table 6). Hence, the PPK model after importing the covariate of body weight was not appropriate. The fitting effect was assessed by the plot of CWRES vs. TAD and logarithmic plots of DV vs. PRED or IPRED, which suggested that the model can appropriately describe the concentration changes in EF in most animals after intravenous administration (Figure 2). The values of V_1_, V_2_, CL, and CL_2_ were estimated to be 0.57 L/kg, 1.28 L/kg, 0.012 L/h/kg, and 1.00 L/h/kg, respectively. The secondary parameter values of K_β_, T_1/2β_, and AUC_0–∞_ were computed to be 0.0065/h, 106.62 h, and 833.33 h.mg/L, respectively.

### 3.4. Model Validation

The bootstrap method was used to validate the final PPK model. The random bootstrap sampling method could randomly generate 1000 sample sets to re-evaluate the initial parameters. The obtained results were compared to the original data sets. The average estimated values were close to the final model results, and the results are shown in Table 4 and Table 6.

## 4. Discussion

The present study is the first to conduct a PPK investigation into EF in largemouth bass after oral gavage and intravenous injection. During the establishment of the PPK model, classic one-compartment, two-compartment, and three-compartment models were used to describe the concentration–time data in largemouth bass after oral and intravenous administrations. We found that the concentration–time data after oral administration were best fitted by the one-compartment model with first-order absorption. However, the two-compartment and three-compartment models could not simulate those data, and the CVs of the parameters were not calculated. As for intravenous administration, the concentration–time data were best fitted by the two-compartment model without absorption. The previous study demonstrated that the immersion phase data of EF were best fitted by the one-compartment model, and injection phase data were best described by the two-compartment model in the PPK studies of sea stars (*Pisaster ochraceus*) [28]. These results were similar to those reported in our studies. In PPK studies on the same class of drug as EF, consistent results were also obtained. It is reported that the plasma data of marbofloxacin in juvenile harbor seals after oral administration were best described by a one-compartment model with first-order input and first-order output [29]. Additionally, Song, Yang, Dai, Zhang, Shao, Wang, Ma, Li and Yang [24] reported a PPK model of danofloxacin in yellow river carp (*Cyprinus carpio haematopterus*) after oral administration, which was also best described by a one-compartment model with first-order absorption.

In the current PPK models, we tried to include the influence of body weight to develop the covariate model. Various equations of body weight affecting the parameters were established under different scenarios. The values of −2LL, AIC, and BIC that were used as the standard were separately calculated out to select the optimal combination. In oral administration, the values of −2LL, AIC, and BIC were the lowest, and the number of parameters was also the lowest in the scenario of V-wt. So, it was introduced in the PPK model to build a covariate model. However, the iteration of the model was not ideal because of no change in the calculated CV in comparison to that in the model without covariates. Using the same method, the optimal combination of covariates was chosen for data following intravenous administration. After simulation, we also found that the alteration of body weight had no significant influence on the iteration of parameters. Subsequently, we evaluated the iteration effect using the plot of CWRES vs. TAD and logarithmic plots of DV vs. PRED or IPRED. Less than 20% of the values were outside the accepted limits of y = 2 and y = 2, indicating that the population pharmacokinetics model adequately described the changes in drug concentrations [30].

After oral administration, the parameter values of K_a_, V, and CL were assessed to be 0.98/h, 6.82 L/kg, and 0.098 L/h/kg, respectively. The secondary parameter values of K_e_, T_1/2Ke_, and AUC_0–∞_ were calculated to be 0.014/h, 49.50 h, and 204.08 h.mg/L, respectively. In traditional PK, a two-compartment model with first-order absorption best described the concentration–time data in largemouth bass after oral administration at 20 mg/kg at 28 °C [17]. In that study, the iterated K_a_ value (10.20/h) was far more than that in the present study; the V_d_ value (2.21 L/kg) was one-third of that in this study, and the CL value (0.017 L/h/kg) was about one-sixth of that in this study. The secondary parameter of T_1/2Ke_ in the present study was about one-half of that (90.79 h) in traditional PK, and the AUC_0–∞_ value was only about one-sixth of that (1185.73 µg.h/mL) in classic PK. These differences may be partly due to the different temperatures used in the separate study. However, the T_1/2Ke_ value and AUC_0–∞_ value at 28 °C were more than those at 25 °C, which may be partly due to the individual variability in the metabolic ability of EF. In allogynogenetic silver crucian carp (*Carassius auratus gibelio*), at 24–26 °C, following oral treatment at 10 mg/kg, the values of T_1/2β_ and AUC_0–∞_ were 62.7 h and 205.9 µg.h/mL [31]. In snakehead fish (*Channa argus*), after oral administration at the same dosage and temperature, the values of T_1/2β_ and AUC_0–∞_ were 35.8 h and 49.98 µg.h/mL [32]. In grass carp receiving oral gavage at a dose of 20 mg/kg at 22 °C, the values of Ka, T_1/2β_, AUC, and CL were 0.67/h, 46.76 h, 171.98 µg.h/mL, and 0.10 L/h/kg, respectively [3]. These values were relatively close to those in our study. In marine fish, the values of T_1/2β_ and AUC_0–∞_ were estimated to be 22.09 h and 54.95 h.mg/L in brown trout (*Salmo truttafario*) following a single oral dose at 10 mg/kg at 10 °C [33]. Additionally, the T_1/2β_ value was evaluated to be 45.22 h in *Takifugu flavidus* receiving a single oral treatment at a dose of 10 mg/kg at 20 °C [34]. The calculated values of T_1/2β_ and AUC were less than those in the current study, which are partly attributable to the salinity promoting the excretion of drugs from fish [4,35].

The values of V and CL were estimated to be 1.85 L/kg and 0.012 L/h/kg after intravenous administration in the present study. The secondary parameter values of K_β_, T_1/2β_, and AUC_0–∞_ were calculated to be 0.0065/h, 106.62 h, and 833.33 h.µg/mL, respectively. In a PPK study on EF in purple sea stars (*Pisaster ochraceus*) after intracelomic injection at a dose of 5 mg/kg at 10–12 °C, values of V, T_1/2β_, and AUC_0–∞_ were all less than the corresponding values in this study [28]. This discrepancy may be due to the different dosages used in the two studies and the disparate ability of metabolism and excretion for the drug in different species of aquatic animals. In traditional PK of EF in allogynogenetic silver crucian carp (*Carassius auratus gibelio*), following a single intravenous dose of 10 mg/kg at 24–26 °C, the T_1/2β_ value (63.50 h) was less than that in the present study and the value of AUC_0–∞_ (239.60 h.µg/mL) was about one-third of that in the current study, but the CL value (0.040 L/h/kg) is about four-fold greater than that in this study [31]. In snakehead fish (*Channa argus*), following the same drug administration and dosage as used in allogynogenetic silver crucian carp, the values of T_1/2β_ (19.82 h) and AUC_0–∞_ (75.79 h.mg/L) were also far less than those in the present study, but the CL value (0.13 L/h/kg) was much greater than that in this study [32]. These shifts may be caused by the different dosages and species variability used in these studies. Additionally, a larger V value (3.40 L/kg) was estimated in brown trout (*Salmo trutta fario*) following intravenous treatment at a dose of 10 mg/kg at 10 °C, suggesting that EF was more extensively distributed in brown trout than in largemouth bass [33]. However, smaller values of T_1/2β_ (19.14 h) and AUC_0–∞_ (70.87 h.mg/L) were calculated in brown trout, which may be attributed to the above-mentioned reason for salinity promoting the excretion of drugs from fish [4,35].

In fish, bioavailability is the speed and amount of drug absorbed into the blood circulation and is an important parameter to evaluate the effectiveness of treatment or optimize a new formulation. In the current study, we also assessed the bioavailability of EF in largemouth bass, finding a value of 12.24%. However, higher values were calculated in allogynogentic silver crucian carp (*Carassius auratus gibelio*, 86.0%), snakehead fish (*Channa argus*, 65.82%), and brown trout (*Salmo trutta fario*, 78.0%). These discrepancies may be attributable in part to different dosage regimens, fish species, temperatures, and other environmental factors in disparate studies.

For fluoroquinolones, pharmacokinetic–pharmacodynamic parameters of AUC/minimum inhibitory concentration (MIC) and C_max_/MIC are imperative indicators to optimize the therapeutic regimens of drugs in humans and animals. It is reported that an indicator of the AUC/MIC value ≥ 125 and C_max_/MIC value ≥ 10 can effectively oppose bacteria to prevent the emergence of drug resistance against fluoroquinolones [36,37]. In pharmacodynamic studies of fish pathogens, the MIC values of EF against *A. hydrophila*, *A. sobria*, *A. salmonicida*, *Y. ruckeri*, and *F. columnare* were 0.063–4.0 µg/mL, 0.063–2.0 µg/mL, 0.004–2.0 µg/mL, 0.004–2.0 µg/mL, and 0.004–0.5 µg/mL, respectively [11,16,38]. The MIC_50_ and MIC_90_ of *F. columnare* were estimated to be 0.016 and 0.125 µg/mL. The MIC_50_ of *A. hydrophila* and *A. sobria* were calculated to be 0.25 and 0.5 µg/mL. In line with the PPK parameters of EF after a single oral dose at 20 mg/kg in this study, the AUC/MIC values were estimated to be 12755.0, 816.32, and 408.16 for *A. hydrophila*, *A. sobria*, and *F. columnare*. These values were far more than the standard value of 125, suggesting the high effectiveness of the present EF regimen for opposing *A. hydrophila*, *A. sobria*, and *F. columnare*.

## 5. Conclusions

This study investigated the PPK parameters of EF in largemouth bass after a single oral dose of 20 mg/kg and a single intravenous dose of 10 mg/kg using sparse sampling collection. A one-compartment model with first-order absorption best described the concentration–time profile of oral administration. The values of K_a_, V, and CL were assessed to be 0.98/h, 6.82 L/kg, and 0.098 L/h/kg, respectively. The secondary parameter values of K_e_, T_1/2Ke_, and AUC_0–∞_ were calculated to be 0.014/h, 49.50 h, and 204.08 h.mg/L, respectively. A two-compartment model without absorption best described the concentration–time profile of intravenous administration. The values of V, V_2_, CL, and CL_2_ were estimated to be 0.57 L/kg, 1.28 L/kg, 0.012 L/h/kg, and 1.00 L/h/kg, respectively. The secondary parameter values of Ke, T_1/2Ke_, and AUC_0–∞_ were calculated to be 0.0065/h, 106.62 h, and 833.33 h.mg/L, respectively. Meanwhile, the AUC/MIC values indicated that EF at 20 mg/kg has high effectiveness for *A. hydrophila*, *A. sobria*, and *F. columnare*. This attempt at a PPK investigation facilitates the development of PK methodology in aquaculture.

## Figures and Tables

**Table 1:** Accuracy and precision of the method for enrofloxacin in spiked plasma in largemouth bass (*Micropterus salmoides*) (n = 5).

Spiked Concentration (μg/mL)	Recovery (%)	Intra-Day RSD (%)	Inter-Day RSD (%)
0.02	103.12 ± 4.32	3.21	6.72
0.1	89.27 ± 4.55	2.07	5.68
1	83.29 ± 2.08	2.11	3.01

Note: RSD, relative standard deviation.

**Table 2:** Initial parameter values of enrofloxacin using a naïve pooled approach in largemouth bass (*Micropterus salmoides*) after oral administration (per os, P.O.) at 20 mg/kg and intravenous administration at 10 mg/kg.

Parameters	Units	P.O.	IV
Ka	/h	1.19	-
V	h	7.30	-
CL	/h	0.086	0.013
V1	h	-	0.51
V2	/h	-	1.31
CL2	L/h/kg	-	1.24

Note: Ka, absorption rate constant for the one-compartment model; V, apparent distribution volume; CL, systemic clearance; V1, apparent distribution volume for central compartment; V2, apparent distribution volume for peripheral compartment; CL2, clearance from central compartment to peripheral compartment; -, not available.

**Table 3:** The calculated values of −2 × log maximum likelihood, the likelihood ratio test, Akaike’s inclusion criteria, and Bayesian information criterion in different scenarios after oral administration, importing the influence of body weight.

Scenarios	LRT	−2LL	AIC	BIC	Parameter (n)
No influence of wt	−84.84	169.67	183.67	200.77	7
Ka-wt	−84.63	169.26	185.26	204.80	8
V-wt	−83.51	167.01	183.01	202.55	8
Ka-wt V-wt	−83.16	166.32	184.32	206.31	9
CL-wt	−84.83	169.67	185.67	205.21	8
Ka-wt CL-wt	−84.59	169.17	187.17	209.16	9
V-wt CL-wt	−83.99	167.98	185.98	207.96	9
Ka-wt V-wt CL-wt	−82.44	164.88	184.88	209.31	10

Note: Ka, absorption rate constant for the one-compartment model; V, apparent distribution volume; CL, systemic clearance; wt, body weight; −2LL, −2 × log maximum likelihood; LRT, likelihood ratio test; AIC, Akaike’s inclusion criteria; BIC, Bayesian information criterion.

**Table 4:** The calculated parameters of enrofloxacin using the population pharmacokinetic model after oral administration.

Scenarios	Parameters	Estimated Values	Units	Standard Error	Coefficient of Variation (%)	2.5% CI	97.5% CI
Importing covariate of wt	tvKa	1.03	/h	0.14	13.49	0.75	1.31
	tvV	6.94	L/kg	0.52	7.45	5.91	7.97
	tvCL	0.097	L/h/kg	0.0091	9.31	0.079	0.12
	dVdwt	−0.39	-	0.17	−43.90	−0.73	−0.049
	stdev0	0.25	-	0.033	13.25	0.18	0.32
Without importing covariate of wt	tvKa	0.98	/h	0.14	14.00	0.71	1.26
	tvV	6.82	L/kg	0.50	7.34	5.82	7.81
	tvCL	0.098	L/h/kg	0.0099	10.16	0.078	0.12
	stdev0	0.30	-	0.046	15.10	0.21	0.39
Bootstrap (n = 1000)	tvKa	1.01	/h	0.16	15.99	0.75	1.37
	tvV	6.90	L/kg	0.54	7.88	5.89	8.02
	tvCL	0.095	L/h/kg	0.013	13.47	0.073	0.12
	stdev0	0.30	-	0.032	10.46	0.24	0.36

Note: tvKa, absorption rate constant with fixed effect; tvV, apparent distribution volume with fixed effect; tvCL, systemic clearance with effect; dVdwt, body weight effect on V; stdev0, residual error; wt, body weight; -, not available.

**Table 5:** The estimated values of −2 × log maximum likelihood (−2LL), the likelihood ratio test (LRT), Akaike’s inclusion criteria (AIC), and Bayesian information criterion (BIC) in different scenarios after intravenous administration, importing the influence of body weight (wt).

Scenarios	LogLik	−2LL	AIC	BIC	Parameter (n)
No influence of wt	−136.35	272.70	290.70	313.30	9.00
V1-wt	−136.05	272.10	292.10	317.21	10.00
V2-wt	−135.17	270.34	290.34	315.45	10.00
V1-wt V2-wt	−135.14	270.28	292.28	319.90	11.00
CL-wt	−136.03	272.07	292.07	317.18	10.00
V1-wt CL-wt	−135.78	271.56	293.56	321.18	11.00
V2-wt CL-wt	−135.13	270.25	292.25	319.87	11.00
V1-wt V2-wt CL-wt	−135.10	270.19	294.19	324.32	12.00
CL2-wt	−135.82	271.65	291.65	316.75	10.00
V1-wt CL2-wt	−135.89	271.78	293.78	321.40	11.00
V2-wt CL2-wt	−134.13	268.25	290.25	317.87	11.00
V1-wt V2-wt CL2-wt	−133.96	267.92	291.92	322.05	12.00
CL-wt CL2-wt	−135.37	270.75	292.75	320.37	11.00
V1-wt CL-wt CL2-wt	−135.44	270.87	294.87	325.00	12.00
V2-wt CL-wt CL2-wt	−134.04	268.08	292.08	322.21	12.00
V1-wt V2-wt CL-wt CL2-wt	−133.85	267.69	293.69	326.33	13.00

Note: V1, apparent distribution volume for central compartment; V2, apparent distribution volume for peripheral compartment; CL, systemic clearance; CL2, clearance from central compartment to peripheral compartment; wt, body weight.

**Table 6:** The estimated parameters of enrofloxacin using the population pharmacokinetic model after oral administration.

Scenarios	Parameter	Estimated Values	Units	Standard Error	Coefficient of Variation (%)	2.5% CI	97.5% CI
Importing covariate of wt	tvV1	0.58	L/kg	0.05	9.23	0.47	0.68
	tvV2	1.25	L/kg	0.09	6.80	1.08	1.42
	tvCL	0.012	L/h/kg	0.00	6.23	0.01	0.01
	tvCL2	0.96	L/h/kg	0.12	12.69	0.72	1.21
	dV2dwt	−0.21	-	0.23	−113.84	−0.67	0.26
	stdev0	0.14	-	0.02	12.57	0.10	0.17
Without importing covariate of wt	tvV1	0.57	L/kg	0.05	9.63	0.46	0.67
	tvV2	1.28	L/kg	0.08	6.55	1.11	1.44
	tvCL	0.012	L/h/kg	0.00	6.12	0.01	0.01
	tvCL2	1.00	L/h/kg	0.12	12.47	0.75	1.25
	stdev0	0.14	-	0.02	11.98	0.11	0.17
Bootstrap (n = 1000)	tvV1	0.54	L/kg	0.095	17.39	0.34	0.70
	tvV2	1.27	L/kg	0.11	8.38	1.09	1.52
	tvCL	0.013	L/h/kg	0.0010	7.88	0.011	0.015
	tvCL2	1.06	L/h/kg	0.21	19.70	0.73	1.52
	stdev0	0.12	-	0.020	17.13	0.079	0.16

Note: tvV1, apparent distribution volume for central compartment with fixed effect; tvV2, apparent distribution volume for peripheral compartment with fixed effect; tvCL, systemic clearance with fixed effect; tvCL2, clearance from central compartment to peripheral compartment with fixed effect; dV2dwt, body weight effect on V2; stdev0, residual error; wt, body weight; -, not available.

## Data Availability

None of the data were deposited in an official repository. The data that support the study findings are available upon request.

## Abbreviations

−2LL, −2 × log maximum likelihood; AIC, Akaike’s inclusion criteria; AUC_0–∞_, area under the plasma concentration vs. time curve from zero to infinity; BIC, Bayesian information criterion; CL, apparent total body clearance per fraction of dose absorbed; CL_2_, apparent clearance from central compartment to peripheral compartment; CV, coefficient of variation; CWRES, conditional weighted residuals; DV, dependent variable; dVdwt, body weight effect on V; dV2dwt, body weight effect on V2; EF, enrofloxacin; HPLC, high-performance liquid chromatography; IPRED, individual predicted concentrations; IV, intravenous administration; K_a_, absorption rate constant for the one-compartment model; K_e_, elimination rate constant for the one-compartment model; K_β_, elimination rate constant for the two-compartment model; K_12_, first-order transport rate constant from central compartment to peripheral compartment; K_21_, first-order transport rate constant from peripheral compartment to central compartment; LRT, likelihood ratio test; MIC, minimum inhibitory concentration; NA, not available; OFV, objective function value; P.O., oral administration (per os); PK, pharmacokinetics; PPK, population pharmacokinetics; RSD, relative standard deviation; stdev0, residual error; t_1/2λz_, terminal half-life; tvCL, systemic clearance with effect; tvCL2, clearance from central compartment to peripheral compartment with fixed effect; tvKa, absorption rate constant with fixed effect; tvV, apparent distribution volume with fixed effect; tvV1, apparent distribution volume for central compartment with fixed effect; tvV2, apparent distribution volume for peripheral compartment with fixed effect; T_1/2β_, elimination half-life in the two-compartmental model; T_1/2Ke_, elimination half-life in the one-compartmental model; TAD, time after dose; T_max_, time to observed maximal concentration after drug administration; V, apparent distribution volume; V_1_, apparent distribution volume for central compartment; V_2_, apparent distribution volume for peripheral compartment; wt, body weight.
